# Traditional Chinese medicine extracts as novel corrosion inhibitors for AZ91 magnesium alloy in saline environment

**DOI:** 10.1038/s41598-022-10900-x

**Published:** 2022-05-05

**Authors:** Haonan Li, Min Fan, Kui Wang, Xiaolan Bian, Haiyan Jiang, Wenjiang Ding

**Affiliations:** 1 National Engineering Research Center of Light Alloy Net Forming, Shanghai Jiao Tong University, Shanghai, 200240 People’s Republic of China; 2 School of Materials Science and Engineering, Shanghai Jiao Tong University, Shanghai, 200240 People’s Republic of China; 3 Department of Pharmacy, Ruijin Hospital, School of Medicine, Shanghai Jiao Tong University, Shanghai, 200003 China

**Keywords:** Engineering, Materials science

## Abstract

*Zingiber officinale* Roscoe extract, *Raphanus sativus* L. extract, *Rheum palmatum* extract, *Coptis chinensis* extract, *Glycyrrhiza uralensis* extract (GUE), *Potentilla* discolor extract (PDE) and *Taraxacum officinale* extract (TOE) were screened for the green corrosion inhibitors of AZ91 alloy in saline environment. The experiment results demonstrated that GUE, PDE and TOE can significantly enhance the corrosion resistance of AZ91 alloy by 73.4, 87.6 and 84.6%, respectively. Surface characterization using FTIR, UV–Vis and XPS revealed that the organic compounds of GUE, PDE and TOE can interact with the alloy surface to form a protective physisorbed film, effectively mitigating the corrosion process of AZ91 alloy. The present results may be helpful to discover the new green inhibitors with high inhibition efficiency for AZ91 alloy.

## Introduction

Magnesium alloys have been utilized extensively as new structural light metal materials in the field of aerospace, automotive, bio-medical, transportation and electronic industries on account of their exceptional properties including high specific strength, high specific stiffness, excellent electromagnetic shielding ability and desirable machinability^1–3^. However, they are intrinsically susceptible to corrosion as the standard equilibrium potential of Mg/Mg^2+^ is − 2.4 V versus SHE^4,5^. Therefore, the principal challenge concerning magnesium alloys is how to improve their corrosion resistance.

According to ASTM-G-15-76 standard, a corrosion inhibitor is a chemical substance or a mixture of several chemical substances that, when added in an appropriate concentration to the corrosive environment, can inhibit the corrosion of metals^6^. In comparison with other anticorrosion techniques such as alloying^7–10^, heat treatment^11–13^ and surface treatment^14–16^, the corrosion control by inhibitors has unique merits such as small addition amounts, low cost and simple operation^17–19^. Currently, corrosion inhibitors can be categorized into three types, i.e. inorganic inhibitors, organic inhibitors and mixtures of inorganic and organic inhibitors, the majority of which are organic compounds with heteroatoms (N, O, S and P) and π bonds in their molecular structure^20^.

Although a number of inhibitors have been developed to effectively mitigate the corrosion of transition metals like Fe and Cu, there is problematic for the application of inhibitors for Mg alloys. Firstly, compared with transition metals, Mg has much less ability to accept π electrons from inhibitor molecules and lone-pair electrons from N, S and O atoms because of its higher energy of 3d orbitals^21,22^. Therefore, the successful inhibitors for other metals may have little or no effect on Mg alloys. Mei et al.^23^, tested 53 bio-relevant organic chemicals on the in vitro corrosion of magnesium and concluded that they can adversely or slightly affect the corrosion rate of magnesium. Secondly, many well-established inhibitors for Mg alloys like phosphates and chromates always have detrimental effects on the environment and human health. Thus, the safety of inhibitors is another concern. Lamaka et al.^24^, investigated the influence of 151 inorganic and organic compounds on the corrosion behavior of Mg and its alloys and found that only 15 compounds can decelerate the corrosion rate, most of which are toxic, carcinogenic and harmful to the environment. Thirdly, a majority of inhibitors for My alloys show poor universality. As pointed by Lamaka et al.^24^, the inhibitors for ZE41 alloy may not be effective in suppressing the degradation of AZ31 alloy. It was reported that only sodium salts of pyridine dicarboxylic acid and salicylic acid derivatives can effectively and universally inhibit the corrosion of pure Mg, Al and RE containing Mg alloys.

To overcome the intrinsic deficiencies of conventional inhibitors, it is imperative to adopt a new strategy to develop novel green and efficient inhibitors, particularly for Mg alloys. In the past few decades, a myriad of studies have been concentrated on the development of eco-friendly inhibitors from natural source. Umoren et al.^25^ screened seven natural polymers including chitosan (CHI), dextran (Dex), carboxymethyl cellulose (CMC), sodium alginate (ALG), pectin (PEC), hydroxylethyl cellulose (HEC) and Gum Arabic (GA) for anti-corrosion effect on AZ31 Mg alloy in saline medium and suggested that CHI, Dex, CMC, PEC and GA exhibited corrosion acceleration effect while HEG and ALG had a moderate inhibition effect with the inhibition efficiency of 64.3 and 58.3%, respectively. In addition to natural polymers, traditional Chinese medicine (TCM) is also derived from green plants and can be deemed as an alternative source of green inhibitors. More significantly, TCM mainly contains heterocyclic rings (e.g. alkaloids, flavonoids and coumarins), strong polar groups such as carboxyl, hydroxyl and phenolic hydroxyl (e.g. quinones, organic acids, saponins and tannins) and long-chain macromolecules (e.g. fatty acids) in their molecular structure. The multiple active centers allow TCM to become a potential candidate for the high-efficient inhibitor. Deyab et al.^26^, found that *Taraxacum officinale* extract (TOE) can be used as a corrosion inhibitor for carbon steel in seawater environment, whose inhibition efficiency was higher than 94%. Ju et al.^27^, studied the corrosion inhibition of hot-dip coated steel in dilute HCl solution by berberine, which was extracted from *Coptis chinensis*. The results revealed that the inhibition efficiency of berberine can reach up to 99.0%. In spite of successful inhibition of steel corrosion upon addition of TCM extracts, to our best of knowledge, there is a scarcity of research on Mg corrosion and few or no TCM extract has been reported as an effective inhibitor for Mg and its alloys. Consequently, it is of vital importance to unveil the effect of TCM extracts on corrosion behavior of Mg alloys.

The present work aims to investigate the anti-corrosion effect on AZ91 Mg alloy of seven TCM extracts, i.e. *Zingiber officinale* Roscoe extract (ZORE), *Raphanus sativus* L. extract (RSLE), Rheum palmatum extract (RPE), *Coptis chinensis* extract (CCE), *Glycyrrhiza uralensis* extract (GUE), *Potentilla* discolor extract (PDE) and *Taraxacum officinale* extract (TOE). The gasometric, electrochemical impedance spectroscopy and potentiodynamic polarization techniques were performed to unravel the degradation behavior of AZ91 alloy in 3.5 wt% NaCl solution with and without TCM extracts. The corrosion surface of AZ91 alloy was analyzed by advanced characterization techniques such as scanning Kelvin probe force microscopy (SKPFM), atomic force microscope (AFM), Fourier-transform infrared spectroscopy (FTIR), ultraviolet–visible spectroscopy (UV–Vis) and X-ray photoelectron spectroscopy (XPS) to clarify the corrosion inhibition mechanism.

## Methods

### The extraction method of TCM

*Zingiber officinale* Roscoe, *Raphanus sativus* L., Rheum palmatum, *Coptis chinensis*, *Glycyrrhiza uralensis*, *Potentilla* discolor and *Taraxacum officinale* were purchased from Leiyunshang Pharmacy of Leiyunshang Pharmaceutical Co., Ltd. (Shanghai, China). The extraction of different targeted compounds was undertaken using the approaches that have been reported in the literature^28–34^. Specifically, the optimal extraction conditions of the supercritical CO_2_ extraction of volatile oil from *Zingiber officinale* Roscoe include the extraction temperature of 35 ℃, the extraction pressure of 35 MPa, the CO_2_ flux of 15 L/h and the extraction time of 2 h. The glycosides were extracted from *Raphanus sativus* L. by ultrasonic extraction method, and the optimal extraction conditions are the ultrasonic power 230 W, extraction time 29 min, ratio of material liquid 1:25 g/mL. The anthraquinones were extracted from *Rheum palmatum* by 75% ethanol through reflux for 5 times and 30 min for each time. The alkaloids were extracted from *Coptis chinensis* by adding 8 times of water at 80 °C for 3 times and 90 min for each time. The optimal extraction conditions of saponin from *Glycyrrhiza uralensis* are the extraction temperature of 50 ℃, the extraction time of 45 min, the ethanol concentration of 60% and the solid–liquid ratio of 1:10. The tannins were extracted from *Potentilla* discolor by 90% ethanol through vacuum reflux and then washed out by 60% ethanol for 15 times. The extraction of polysaccharides from *Taraxacum officinale* was performed by ultrasonic extraction method, and the optimal extraction parameters are the ultrasonic time of 63 min, the ultrasonic temperature of 73 °C, the ultrasonic power of 120 W, and the liquid to material ratio of 25:1 mL/g.

### AZ91 alloy preparation

AZ91 Mg alloy were produced by melting high pure magnesium (99.9% purity), aluminum (99.5%) and zinc (99.9%) in a graphite crucible at 720 °C under the protection of mixed gas of CO_2_/SF6. After the removal of gases and inclusions using the mixture of MgCl_2_, KCl, BaCl_2_ and CaF_2_ (2.0 wt%) at 740 °C, the melts were poured into a cylindrical permanent mold preheated to 300 °C. All samples used for corrosion test were sectioned from the center of ingots and machined into the cuboids with size of 1 cm × 1 cm × 0.5 cm. The samples were all grounded with emery-paper of #300, #1500, #3000 and #7000 grits and then rinsed in ethanol with ultrasonication assistance. Note that three samples were applied for each test in corrosion performance measurements to achieve good reproducibility.

### Solution preparation

The 3.5 wt% NaCl solution was synthesized by proportionally dissolving NaCl salt into the distilled water. The seven TCM extracts including ZORE, RSLE, RPE, CCE, GUE, PDE and TOE with the concentration of 1.0 g/L were respectively added into 3.5 wt% NaCl solution for the anticorrosion performance screening. The blank one that without any TCM extract was also investigated for comparison. Besides, the screened TCM extracts with different addition levels of 0.5, 1.0, 2.0 and 2.5 g/L were also added into 3.5 wt% NaCl solution to investigate the effect of addition level on the inhibition performance.

### Hydrogen evolution

Hydrogen evolution tests were carried out on 1 cm^2^ area of AZ91 sheet using eudiometers. The samples were inlaid in epoxy resin with a surface exposed. All tests were performed for 24 h at ambient temperature. The degradation rate (v_d_, cm^3^ h^−1^) was calculated using the equation given by^35^

1 $$ {\text{v}}_{{\text{d}}} = \frac{{{\text{V}}_{{\text{t}}} - {\text{V}}_{{\text{i}}} }}{{{\text{t}}_{{\text{t}}} - {\text{t}}_{{\text{i}}} }} $$

where V_t_ and V_i_ are the volumes of gas released at time t_t_ and t_i_, respectively.

### Weight loss experiments

The weight loss experiments were performed on matrix alloy and AZ91 nanocomposites. All samples were pre-weighed and then immersed into the isolated cylindrical glass beaker filled with 3.5 wt% NaCl solution without and with screened TCM extracts for 24 h at atmospheric temperature. The corroded samples after immersion were cleaned in 200 g/l CrO_3_ and 10 g/l AgNO_3_^36^, rinsed with de-ionized water and dried with hot air and then weighed again. The weight loss was recorded at the duration time of 8, 16 and 24 h. The corrosion rate ${\text{V}}_{{{\text{wl}}}}$ (mm/year) examined by weight loss is calculated by given equation^37^:

2 $$ {\text{v}}_{{{\text{wl}}}} = 8.74 \times 10^{4} \times \frac{{{\text{m}}_{1} - {\text{m}}_{2} }}{{{\text{A}}_{{\text{s}}} \cdot {\text{t}} \cdot {\text{d}}}} $$

where ${\text{m}}_{1}$ and ${\text{m}}_{2}$ is the sample mass before and after immersion, respectively. ${\text{A}}_{{\text{s}}}$ (cm^2^) is the surface area of samples, ${\text{t}}$ (h) is the duration time and ${\text{d}}$ (g/cm^3^) is the density of alloy. Note that the exposed surface area of 4 cm^2^ of samples were adopted for weight loss tests in this work.

### Electrochemical experiments

A Princeton Electrochemical Workstation (PARSTAT4000A) was used for the electrochemical impedence spectroscopy (EIS) and the potentiodynamic polarization (PDP) experiments. The experimental instrument is composed of a three-electrode cell, AZ91 alloy sample as a working electrode, a saturated KCl electrode as a reference electrode and a platinum sheet as a counter electrode. The test area of all samples is 1 cm^2^. The open circuit potential (OCP) measurement was performed for 1800s to obtain a pseudo steady state condition before ESI experiments. The EIS tests were performed in the frequency range of 100,000 Hz to 0.1 Hz with AC amplitude of 10 mV at open circuit potential (OCP). The PDP experiments were carried out at the potential of ± 0.3 V versus OCP with a scan rate of 0.1 mV s^−1^.

### SEM, LCM, XPS, FTIR, UV–Vis, SKPFM experiments

The surface observation of AZ91 alloy samples before and after immersing in 3.5 wt% NaCl solutions for 24 h with and without TCM extracts was performed using a TESCAN-RISE scanning electron microscopy (SEM). The voltages of 5 and 15 kV were adopted for morphology observation and EDS mapping, respectively.

A Laser Confocal microscopy (LCM, VK-X3000) was used to characterize the morphology and roughness of exposed surface of AZ91 samples after immersing in 3.5 wt% NaCl solution with and without TCM extracts for 24 h. AXIS UltraDLD XPS spectrometer was used to analyze the information of elements and their valence states of corroded surface of AZ91 samples.

The Fourier Transform infrared spectroscopy (FTIR) analysis was conducted for the screened TCM extracts and the surface of corroded AZ91 alloy samples immersed in 3.5 wt% NaCl solution containing the screened TCM extracts for 24 h at 25 ℃. The FTIR spectra were recorded in the range of 350–12,000 cm^−1^ using a FTIR spectrophotometer (Thermo Scientific Nicolet iN10 MX, America). A Lamda 950 UV–Vis spectrophotometer was utilized to record the UV–Vis spectra of 3.5 wt% NaCl solution with and without the screened TCM extracts after immersion of AZ91 alloy samples for 24 h. The UV–Vis scanning was performed in the range of 175–3300 nm.

A scanning Kelvin probe force microscopy (SKPFM, Dimension Fastscan Bio, Bruker) was adopted to measure the Volta potential difference between β-Mg_17_Al_12_ and α-Mg of AZ91 alloy samples immersed in 3.5 wt% NaCl solution containing the screened TCM extracts for 24 h. The uncorroded AZ91 sample was also measured for comparison. All tests were carried out at ambient temperature. The scanning area was 20 μm × 20 μm. The SKPFM data was analyzed using NanoScope analysis software.

### Plants statement

The seven plant extracts used in this work comply with IUCN Policy Statement on Research Involving Species at Risk of Extinction and the Convention on the Trade in Endangered Species of Wild Fauna and Flora.

## Results

### Screening of TCM extracts

**Figure 1:**
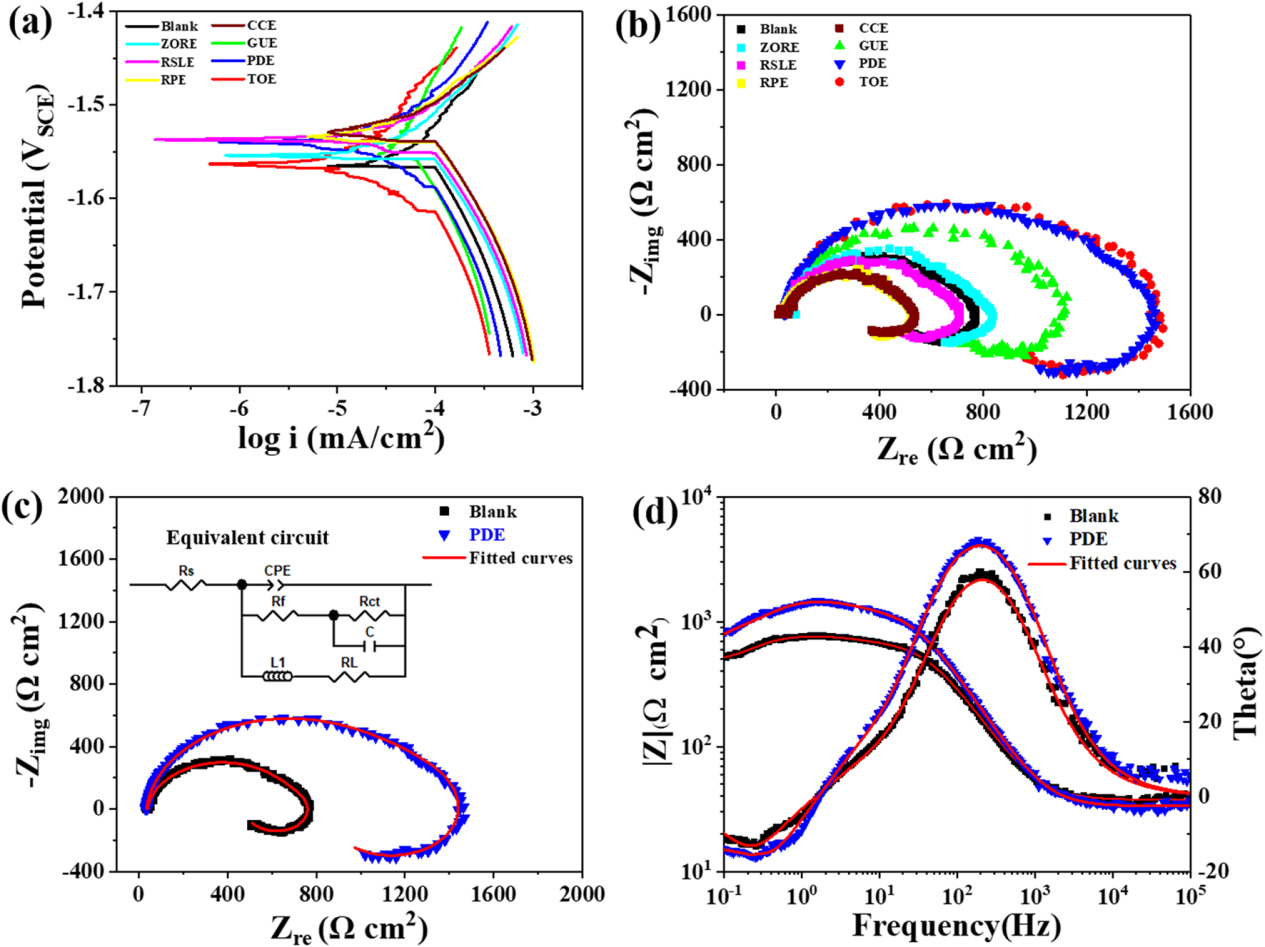
Electrochemical tests of AZ91 Mg alloy in 3.5 wt% NaCl solution without and with seven 1 g/L TCM extracts. (**a**) Potentiodynamic polarization curves; (**b**) Electrochemical impedance spectra; comparisons between original data and fitted results of (**c**) EIS curves and (**d**) bode diagrams of the AZ91 alloy and a typical AZ91alloy upon the addition of PDE.

Seven TCM extracts including ZORE, RSLE, RPE, CCE, GUE, PDE and TOE were screened for the inhibition effect towards AZ91 alloy corrosion in 3.5 wt% NaCl solution by electrochemical experiments. The results were illustrated in Fig. 1, Tables 1 and 2. Figure 1a displays the polarization curves of AZ91 alloy in 3.5 wt% NaCl solution without and with addition of TCM extracts. In principle, the high corrosion potential and the low corrosion current density mean the good inhibition performance. The tafel curve is characterized by the anodic and cathodic branches, which are associated with Mg dissolution and hydrogen evolution, respectively. It is apparent that the corrosion current density shifted negatively and the corrosion reaction was suppressed after addition of GUE, PDE and TOE. Based on the PDP results, the inhibiting efficiency values (${\upeta }_{\mathrm{Icorr}}$) of GUE, PDE and TOE are estimated to be 47.14, 66.90 and 58.21% as listed in Table 1, respectively.

**Table 1:** Electrochemical parameters obtained from the polarization curves of AZ91 Mg alloy in 3.5 wt% NaCl solution without and with addition of 1 g/L seven TCM extracts at normal temperature.

System	β_a_ (mV/dec)	β_c_ (mV/dec)	−Ecorr (mV )	Icorr (μA cm^−2^)	*η*_Icorr_ (%)
Blank	223 ± 12	196.9 ± 9.6	1565 ± 9	104.69 ± 3.22	–
ZORE	184 ± 9	170.7 ± 6.3	1551 ± 7	92.36 ± 2.31	11.77
RSLE	197.9 ± 3.2	224.1 ± 3.3	1536 ± 6	111.35 ± 1.97	− 6.36
RPE	152.2 ± 4.5	210.8 ± 4.6	1533 ± 3	124.37 ± 2.73	− 18.80
CCE	162.2 ± 3.9	231.7 ± 5.3	1525 ± 5	122.12 ± 2.24	− 16.65
GUE	143.8 ± 5.1	167 ± 6	1553 ± 4	55.34 ± 1.02	47.14
PDE	136.7 ± 3.3	151.9 ± 4.7	1540 ± 6	34.65 ± 0.77	66.90
TOE	175 ± 6	169.8 ± 5.8	1562 ± 5	43.75 ± 1.15	58.21

**Table 2:** Corresponding fitted parameters of EIS spectra of AZ91 Mg alloy in 3.5 wt% NaCl solution without and with addition of 1 g/L seven TCM extracts at normal temperature.

System	R_S_ (Ω cm^2^)	R_f_ (Ω cm^2^)	R_ct_ (Ω cm^2^)	CPE (10^–6^ S s^n^ cm^−2^)	n	C (10^–4^ F)	L_1_ (Ω cm)	RL_1_ (Ω cm^2^)	R_P_ (Ω cm^2^)	η_EIS_ (%)
Blank	27.33 ± 1.23	607.36 ± 5.32	32.77 ± 2.12	9.88 ± 0.12	0.91 ± 0.01	4.39 ± 0.01	1005.5 ± 6.3	449.29 ± 3.21	263.91 ± 3.12	–
ZORE	34.86 ± 2.15	613.58 ± 6.87	41.75 ± 3.45	8.04 ± 0.09	0.88 ± 0.02	5.61 ± 0.03	832.5 ± 4.3	452.35 ± 2.95	267.57 ± 2.97	1.68
RSLE	19.66 ± 1.67	654.82 ± 5.12	26.38 ± 2.09	6.76 ± 0.06	0.91 ± 0.01	6.14 ± 0.01	586.2 ± 3.8	423.11 ± 3.17	261.08 ± 2.53	− 0.75
RPE	28.71 ± 2.65	473.86 ± 4.95	20.33 ± 1.38	9.56 ± 0.15	0.88 ± 0.02	12.17 ± 0.05	456.6 ± 3.4	494.97 ± 2.83	247.26 ± 2.28	− 6.36
CCE	41.88 ± 3.64	470.82 ± 3.45	22.79 ± 1.27	6.88 ± 0.07	0.88 ± 0.03	7.09 ± 0.02	332.2 ± 2.9	445.13 ± 2.81	234.07 ± 2.34	− 12.37
GUE	27.86 ± 2.99	862.35 ± 6.31	105.14 ± 3.63	7.73 ± 0.07	0.90 ± 0.01	1.49 ± 0.01	934.3 ± 4.2	1188.62 ± 5.17	533.18 ± 3.72	50.66
PDE	33.12 ± 2.13	1362.64 ± 9.31	167.42 ± 3.31	9.28 ± 0.14	0.89 ± 0.02	1.40 ± 0.01	1164 ± 5.1	1810.68 ± 5.33	828.99 ± 4.36	68.27
TOE	41.63 ± 3.15	1310.46 ± 8.95	139.12 ± 3.97	8.37 ± 0.11	0.93 ± 0.01	1.80 ± 0.01	1371 ± 6.1	1912.35 ± 5.73	824.43 ± 6.21	68.09

Figure 1b shows the Nyquist plots for different TCM extracts. It is observable that the larger radius of Nyquist semicircle was achieved upon addition of GUE, PDE and TOE in comparison with other TCM extracts, indicating the better corrosion protection. Figure 1c,d demonstrate the comparisons between original data and fitted results of EIS curves and bode diagrams of the AZ91 alloy and a typical AZ91alloy upon the addition of PDE by using the given equivalent circuit model in inset of Fig. 1c, respectively. The capacitance C is introduced due to the fact that the corresponding n value is equal to 1.The excellent overlap between fitted curves and original data ensures the veracity of the used equivalent circuit, in which the charge transfer resistance (${\text{R}}_{{{\text{ct}}}}$), the inductive resistance (${\text{R}}_{{\text{L}}}$) and the film resistance (${\text{R}}_{{\text{f}}}$) are involved. In this work, the polarization resistance (${\text{R}}_{{\text{p}}}$) can be simplified as

3 $$ \frac{1}{{{\text{R}}_{{\text{P}}} }} = \frac{1}{{{\text{R}}_{{\text{f}}} + {\text{R}}_{{{\text{ct}}}} }} + \frac{1}{{{\text{R}}_{{\text{L}}} }} $$

The inhibiting efficiency can be determined by Eqs. (4) and (5)

4 $$ {\upeta }_{{{\text{EIS}}}} = \left( {\frac{{{\text{R}}_{{{\text{PEIS}}\left( 1 \right)}} - {\text{R}}_{{{\text{PEIS}}\left( 0 \right)}} }}{{{\text{R}}_{{{\text{PEIS}}\left( 1 \right)}} }}} \right) \times 100{\text{\% }} $$

5 $$ {\upeta }_{{{\text{Icorr}}}} = \left( {\frac{{{\text{I}}_{{{\text{corr}}\left( 0 \right)}} - {\text{I}}_{{{\text{corr}}\left( 1 \right)}} }}{{{\text{I}}_{{{\text{corr}}\left( 0 \right)}} }}} \right) \times 100{\text{\% }} $$

where ${\text{R}}_{{{\text{PEIS}}\left( 1 \right)}}$ and ${\text{R}}_{{{\text{PEIS}}\left( 0 \right)}}$ are the total resistance with and without TCM extracts, respectively. ${\text{ I}}_{{{\text{corr}}\left( 1 \right)}}$ and ${\text{I}}_{{{\text{corr}}\left( 0 \right)}}$ are the corrosion current density with and without TCM extracts, respectively. The calculated values of ${\text{R}}_{{{\text{PEIS}}}}$, ${\upeta }_{{{\text{EIS}}}}$(%) and ${\upeta }_{{{\text{Icorr}}}}$(%) are summarized in Table 2. From Table 2, it can be seen that GUE, PDE and TOE can inhibit the corrosion of AZ91 alloy, whereas the others can accelerate or slightly decelerate the corrosion rate. For the addition level of 1.0 g/L, the ${\upeta }_{{{\text{EIS}}}}$(%) values of GUE, PDE and TOE can reach 50.66%, 68.27% and 68.09%, respectively, which are basically consistent with the PDP results. Therefore, it can be concluded that GUE, PDE and TOE can exhibit a moderate inhibiting effect, among which PDE has the highest efficiency.

**Figure 2:**
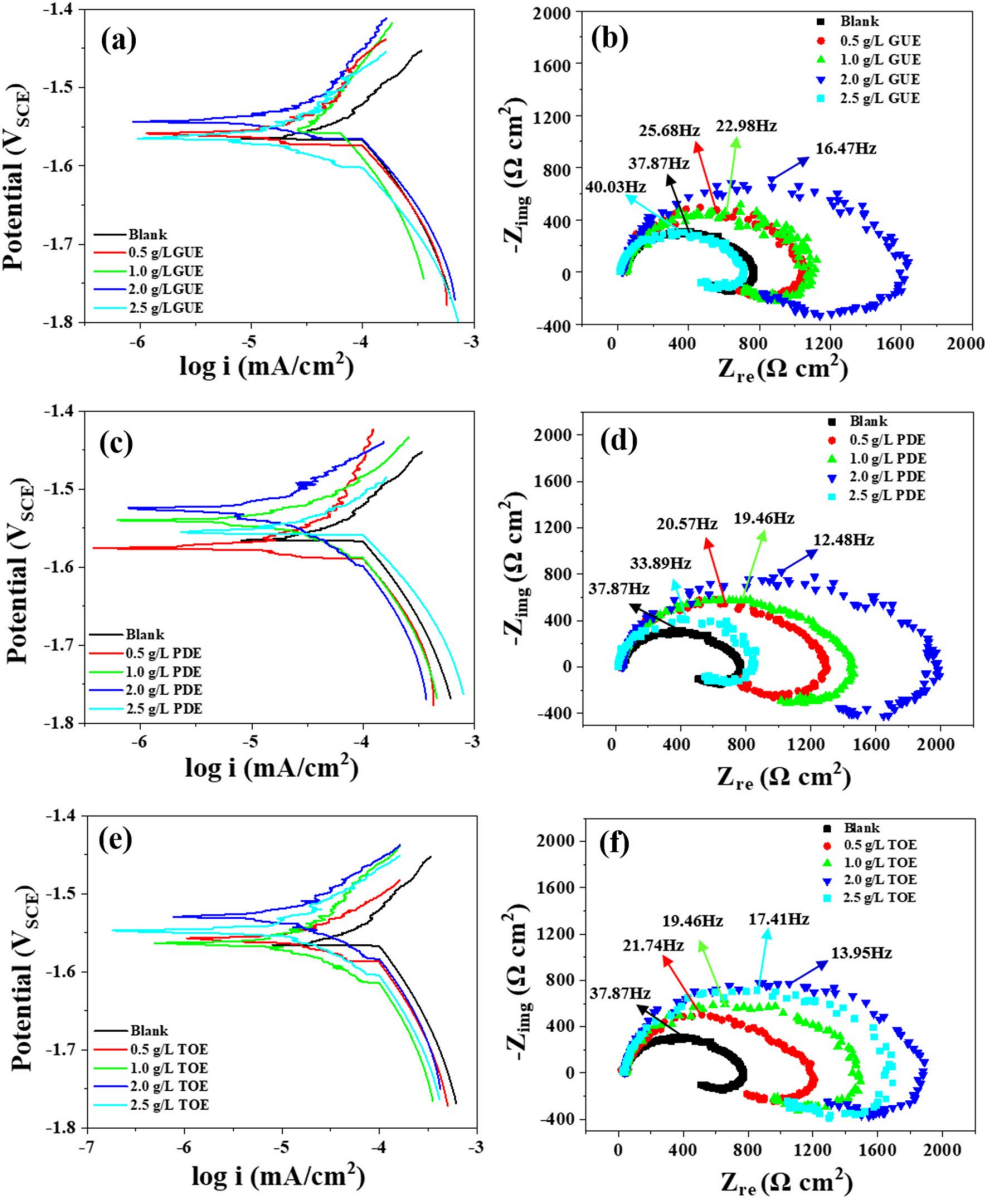
Electrochemical characteristics of AZ91 Mg alloy in 3.5 wt% NaCl solution without and with different concentrations of (**a**,**b**) GUE, (**c**,**d**) PDE, (**e**,**f**) TOE at normal temperature.

According to the preliminary screening results, it is of great necessity to clarify the concentration effect on the inhibition property of GUE, PDE and TOE. As a result, a wide range of addition levels from 0.5 to 2.5 g/L were taken into account during AZ91 alloy corrosion studies. The electrochemical analysis was performed as a function of concentration of GUE, PDE and TOE and the results were plotted as Tafel and Nyquist curves in Fig. 2. The obtained polarization and impedance parameters were given in Tables 3 and 4. In general, it can be found that the polarization current density was progressively decreased with increasing the concentration of GUE, PDE and TOE from 0.5 to 2.0 g/L and then significantly increased when the concentration reached 2.5 g/L. The polarization resistance and the inhibiting efficiency (${\upeta }_{{{\text{EIS}}}}$ and ${\upeta }_{{{\text{Icorr}}}}$) show an opposite variation trend, i.e. a gradual increase till 2.0 g/L, afterwards a sharp reduction. It is well-documented that the organic inhibitors can yield the inhibition effect mainly by their adsorption onto the metal surface^36^. Nevertheless, the inhibitor at a low concentration e.g. 0.5 g/L may fail to induce an adequate adsorption. In addition, for the excessive concentration of inhibitors, e.g. 2.5 g/L, would cause a decrease in inhibition performance compared with that of 2 g. Actually, the number of molecules would be significantly increased as the concentration increases, leading to a shorter distance between individual molecules. As a result, the molecules aggregation would be aggravated. It is inevitable that the adsorbed molecules could also interact with the un-adsorbed molecules and result in the separation of adsorbed molecules from the metal surface. Once the detachment of adsorbed molecules occurs, the exposed surface would be corroded. thus lead to the weakening of corrosion inhibition. The similar phenomena have been reported in the corrosion inhibition of mild steel by benzisothiozole-3-piperizine hydrochloride, 5-(benzo[d][1,3]dioxol-5-ylmethyl)-2-tetradecyl isoxazolidine and a diallylmethylamine-based cyclopolymer^38–40^. When the concentration of inhibitors reaches 2.0 g/L, the surface coverage may be maximized to afford the most remarkable inhibition effect owing to the adsorption of sufficient inhibitor molecules. Therefore, as far as the corrosion inhibition of AZ91 alloy in 3.5 wt% NaCl solution is concerned, 2.0 g/L can be deemed as the optimal addition level for GUE, PDE and TOE.

**Table 3:** Corresponding fitted parameters of PDP curves and EIS spectra of AZ91 Mg alloy in 3.5 wt% NaCl solution without and with different concentrations of GUE, PDE, TOE at normal temperature.

System	R_S_ (Ω cm^2^)	R_f_ (Ω cm^2^)	R_ct_ (Ω cm^2^)	CPE (10^–6^ S s^n^ cm^−2^)	n	C (10^–4^ F)	L_1_ (Ω cm)	RL_1_ (Ω cm^2^)	R_P_ (Ω cm^2^)	η_EIS_ (%)
Blank	27.33 ± 1.23	607.36 ± 5.32	32.77 ± 2.12	9.88 ± 0.12	0.91 ± 0.01	4.39 ± 0.01	1005.5 ± 6.3	449.29 ± 2.12	263.91 ± 3.12	**−**
GUE-0.5 g/L	31.36 ± 2.01	929.85 ± 7.31	27.99 ± 1.81	8.04 ± 0.09	0.88 ± 0.02	7.04 ± 0.02	542.7 ± 2.5	1147.21 ± 5.94	553.35 ± 4.57	49.61
GUE-1.0 g/L	27.86 ± 2.99	862.35 ± 6.31	105.14 ± 3.63	7.73 ± 0.07	0.90 ± 0.01	1.49 ± 0.01	934.3 ± 4.2	1188.62 ± 6.01	533.18 ± 3.72	50.66
GUE-2.0 g/L	27.37 ± 1.01	1328.12 ± 8.64	288.24 ± 2.01	9.56 ± 0.15	0.88 ± 0.02	1.34 ± 0.00	1009 ± 4.5	2127.54 ± 8.21	945.71 ± 4.81	71.36
GUE-2.5 g/L	13.69 ± 0.95	604.31 ± 3.09	46.28 ± 3.06	6.88 ± 0.07	0.88 ± 0.03	3.80 ± 0.01	844.2 ± 3.4	534.71 ± 3.21	307.07 ± 2.07	10.33
PDE-0.5 g/L	26.95 ± 1.5	1208.54 ± 5.62	70.65 ± 3.17	7.73 ± 0.07	0.90 ± 0.01	3.86 ± 0.01	840.2 ± 3.6	1438.03 ± 5.94	703.81 ± 4.28	61.14
PDE-1.0 g/L	33.12 ± 2.13	1362.64 ± 9.31	167.42 ± 3.31	9.28 ± 0.14	0.89 ± 0.02	1.40 ± 0.01	1164.3 ± 5.1	1810.68 ± 6.31	828.99 ± 4.36	68.27
PDE-2.0 g/L	38.59 ± 2.64	1654.21 ± 9.54	380.64 ± 2.13	8.37 ± 0.11	0.93 ± 0.01	1.11 ± 0.00	1623.2 ± 5.9	4904.53 ± 11.23	1476.65 ± 6.33	81.71
PDE-2.5 g/L	13.29 ± 0.76	731.97 ± 5.83	51.25 ± 1.16	7.75 ± 0.07	0.88 ± 0.01	2.85 ± 0.02	660.7 ± 2.8	587.07 ± 4.21	348.81 ± 1.79	21.61
TOE-0.5 g/L	44.41 ± 3.03	1033.21 ± 6.67	120.18 ± 2.12	8.76 ± 0.07	0.90 ± 0.01	4.18 ± 0.02	1653.1 ± 6.7	1324.83 ± 5.31	660.72 ± 2.31	57.33
TOE-1.0 g/L	41.63 ± 3.15	1310.46 ± 8.95	139.12 ± 3.97	8.37 ± 0.11	0.93 ± 0.01	1.80 ± 0.01	1371.7 ± 6.1	1912.35 ± 6.85	824.43 ± 6.21	68.09
TOE-2.0 g/L	38.94 ± 2.95	1502.47 ± 9.95	346.67 ± 2.11	6.95 ± 0.07	0.88 ± 0.03	1.64 ± 0.00	2553.8 ± 7.1	4013.73 ± 9.31	1304.57 ± 9.06	79.22
TOE-2.5 g/L	37.89 ± 2.56	1514.35 ± 8.91	206.18 ± 0.98	7.86 ± 0.07	0.90 ± 0.01	2.09 ± 0.03	1177.9 ± 5.1	1973.29 ± 5.12	956.81 ± 7.35	71.38

**Table 4:** Electrochemical parameters obtained from the polarization curves of AZ91 Mg alloy in 3.5 wt% NaCl solution without and with different concentrations of GUE, PDE, TOE at normal temperature.

System	β_a_ (mV/dec)	β_c_ (mV/dec)	−Ecorr (mV)	Icorr (μA cm^−2^)	*η*_Icorr_ (%)
Blank	223 ± 12	196.9 ± 9.6	1565 ± 9	104.69 ± 3.22	–
GUE-0.5 g/L	183.3 ± 7.9	132.3 ± 5.6	1544 ± 7	62.56 ± 1.34	40.24
GUE-1.0 g/L	143.8 ± 5.1	167 ± 6	1553 ± 4	55.34 ± 1.02	47.14
GUE-2.0 g/L	124.4 ± 6.2	89.3 ± 3.9	1542 ± 3	27.85 ± 0.97	73.40
GUE-2.5 g/L	153.3 ± 9.1	153.3 ± 5.9	1552 ± 4	84.32 ± 2.83	19.46
PDE-0.5 g/L	147.9 ± 7.6	120.9 ± 7.1	1573 ± 9	41.03 ± 1.85	60.81
PDE-1.0 g/L	136.7 ± 3.3	151.9 ± 4.7	1540 ± 6	34.65 ± 0.77	66.90
PDE-2.0 g/L	76.3 ± 2.1	95 ± 7	1520 ± 5	13.01 ± 0.13	87.57
PDE-2.5 g/L	146.3 ± 6.3	153.9 ± 6.4	1552 ± 7	75.32 ± 2.16	28.05
TOE-0.5 g/L	139.3 ± 4.9	161.4 ± 8.1	1554 ± 6	50.69 ± 1.67	51.58
TOE-1.0 g/L	175 ± 6	169.8 ± 5.8	1562 ± 5	43.75 ± 1.15	58.21
TOE-2.0 g/L	97.9 ± 2.9	71.7 ± 6.5	1529 ± 7	16.13 ± 0.27	84.59
TOE-2.5 g/L	139.2 ± 3.1	161.1 ± 3.3	1548 ± 5	33.75 ± 1.03	67.76

Figure 2a is the polarization curves of AZ91 alloy in 3.5 wt% NaCl solution containing various concentrations of GUE. Compared with the blank, the anodic and cathodic branches shifted to lower current region. As the concentration increased, the value of corrosion potential shifted toward positive direction, revealing that GUE is an anodic-type inhibitor^41,42^. As shown in Table 4, with increasing the concentration of GUE to 2.0 g/L, the corrosion current density was greatly decreased from 104.69 ± 3.22 to 27.85 ± 0.97 μA cm^−2^ and the corrosion potential was increased form − 1565 ± 9 to − 1542 ± 3 mV. The inhibiting efficiency ${\upeta }_{{{\text{Icorr}}}}$ was significantly increased to 73.4%. Figure 2c shows the polarization curves of AZ91 alloy in 3.5 wt% NaCl solution in the presence of PDE of different concentrations. Similarly to GUE, the addition of PDE at the concentration ranging from 0.5 to 2.0 g/L could result in the shift of polarization curves to lower current region and corrosion potential increased. Thus, PDE can act as an anodic-type inhibitor. The increase of PDE concentration from 0.5 to 2.0 g/L caused the decrease of current density from 41.03 ± 1.85 to 13.01 ± 0.13 μA cm^−2^ and the increase of corrosion potential from − 1573 ± 9 to 1520 ± 5 mV. The ${\upeta }_{{{\text{Icorr}}}}$ increased from 60.8 to 87.6%. Figure 2e presents the polarization curves of AZ91 alloy in 3.5 wt% NaCl solution with addition of various concentrations of TOE. As the concentration was increased, the shift of the potential (Ecorr) was rather negligible. According to the literature^43^, if the displacement in Ecorr is < 85 mV with respect to Ecorr, the inhibitor can be seen as a mixed type. From the Table 4, it can be seen that the difference in Ecorr between blank solution and with the addition of TOE at different concentration are all less than 85 mV. Therefore, the TOE may be considered as a mixed-typed inhibitor. When the concentration was raised up to 2.0 g/L, the corrosion current density was minimized to 16.13 ± 0.27 μA cm^−2^ , corrosion potential was maximized to 1529 ± 7 mV and the ${\upeta }_{{{\text{Icorr}}}}$ was maximized to 84.6%.

Figure 2b,d,f show the variations of electrochemical impedance spectra with the concentration of GUE, PDE and TOE, respectively. The increased level of GUE, PDE and TOE addition could give rise to an increased size of Nyquist semicircle and therefore a more pronounced corrosion protection. For GUE, as the concentration reached 2.0 g/L, the polarization resistance was increased from 263.91 ± 3.12 to 945.71 ± 4.81 Ω cm^2^ and the inhibiting efficiency ${\upeta }_{\mathrm{EIS}}$ was raised to 71.4%. For PDE, the addition level of 2.0 g/L can lead to the increment of polarization resistance to 1476.65 ± 6.33 Ω cm^2^ and the ${\upeta }_{\mathrm{EIS}}$ to 81.7%. For TOE, the increased polarization resistance was accompanied by the increased inhibiting efficiency with the increase of addition level. The highest values of polarization resistance and ${\upeta }_{\mathrm{EIS}}$ were 1304.57 ± 9.06 Ω cm^2^ and 79.2%, respectively, at the concentration of 2.0 g/L. By comparison, it is evident that the PDP results are in good agreement with those of EIS.

**Figure 3:**
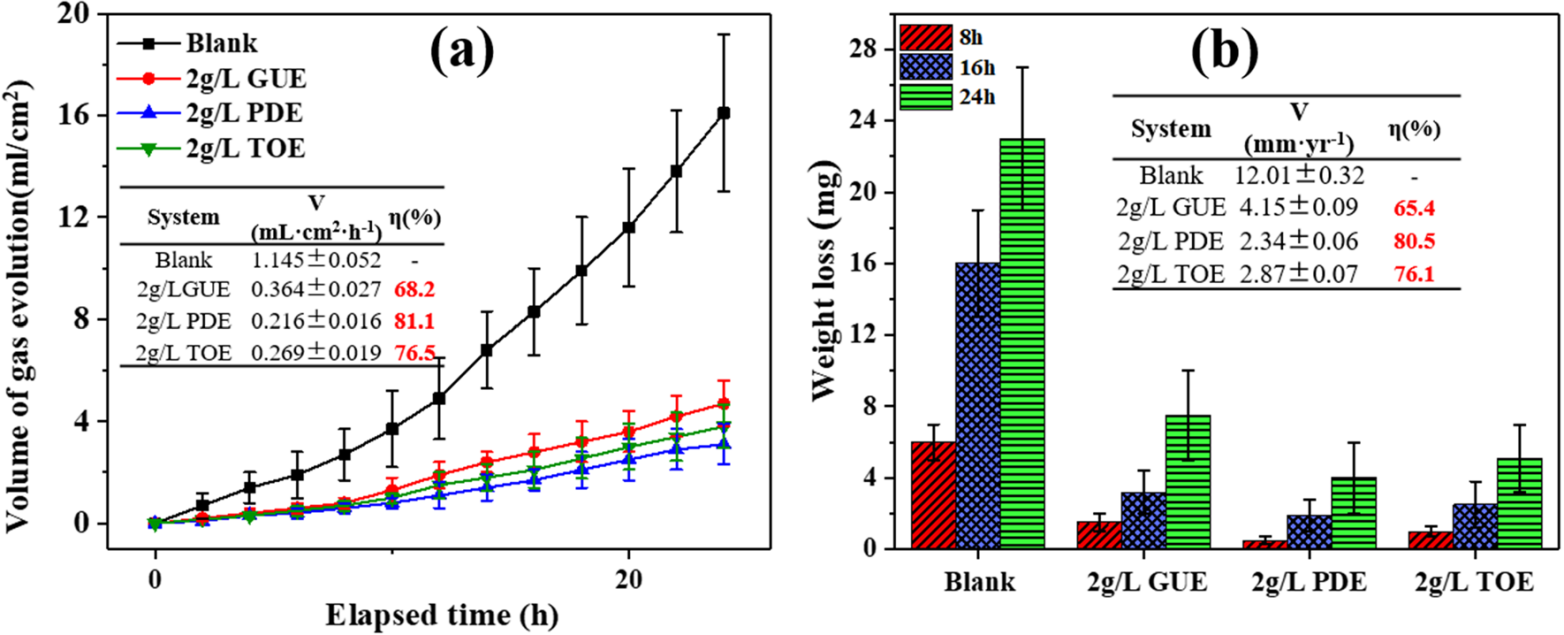
Immersion tests of AZ91alloys in 3.5 wt% NaCl solution without and with 2.0 g/L GUE, PDE and TOE for 24 h. (**a**) Hydrogen evolution volume as function of elapsed time; (**b**) Wight-loss at different time intervals.

The dependence of hydrogen evolution on time during AZ91 alloy corrosion in 3.5 wt% NaCl solution without and with 2.0 g/L GUE, PDE and TOE is illustrated in Fig. 3a. From the H_2_ volume-time curves, it can be seen that H_2_ production presented a nearly linear variation with time. The volume of H_2_ evolution for 24 h was recorded and used for the evaluation of inhibiting efficiency. According to the hydrogen evolution results, the inhibiting efficiency (${\upeta }_{{{\text{H}}_{2} }}$) of inhibitors indicated by hydrogen evolution can be defined as

6 $$ {\upeta }_{{{\text{H}}_{2} }} = \left( {\frac{{{\text{V}}_{{\text{d}}}^{0} - {\text{V}}_{{\text{d}}}^{{{\text{Inh}}}} }}{{{\text{V}}_{{\text{d}}}^{0} }}} \right) \times 100{\text{\% }} $$

where ${\text{V}}_{{\text{d}}}^{0}$ and ${\text{V}}_{{\text{d}}}^{{{\text{Inh}}}}$ are the amounts (mL) of H_2_ evolution for AZ91 sample immersed in 3.5 wt% NaCl solution without and with 2.0 g/L GUE, PDE and TOE, respectively. It is evident that the hydrogen evolution caused by the alloy degradation was markedly stifled upon addition of GUE, PDE and TOE. Specifically, the rate of gas evolution was 1.145 ± 0.052 mL cm^−2^ h^−1^ in the blank solution. After addition of 2.0 g/L GUE, PDE and TOE to the corrosive medium, it was reduced to 0.364 ± 0.027 mL cm^−2^ h^−1^, 0.216 ± 0.016 mL cm^−2^ h^−1^ and 0.269 ± 0.019 mL cm^−2^ h^−1^, respectively. In the presence of GUE, PDE and TOE, the gas evolution was much lesser than that of the blank, and the inhibiting efficiency ${\upeta }_{{{\text{H}}_{2} }}$ was determined to be 68.2, 81.1 and 76.5%, respectively. In addition, the weight-loss experiments in Fig. 3b reveal that the smaller reduction in weight-loss can be achieved upon the addition of GUE, PDE and TOE compared with blank. The inhibition efficiency (${\upeta }_{{{\text{wl}}}}$) evaluated by weight-loss can be defined as

7 $$ {\upeta }_{{{\text{wl}}}} = \left( {\frac{{{\text{V}}_{{{\text{wl}}}}^{0} - {\text{V}}_{{{\text{wl}}}}^{{{\text{Inh}}}} }}{{{\text{V}}_{{{\text{wl}}}}^{0} }}} \right) \times 100{\text{\% }} $$

where ${\text{V}}_{{{\text{wl}}}}^{0}$ and ${\text{V}}_{{{\text{wl}}}}^{{{\text{Inh}}}}$ are the weight loss corrosion rate of AZ91 sample immersed in 3.5 wt% NaCl solution without and with 2.0 g/L GUE, PDE and TOE. It can be seen that the inhibiting efficiency ${\upeta }_{{{\text{wl}}}}$ was determined to be 65.4, 80.5 and 76.1% for the GUE, PDE and TOE, respectively. The good agreement of hydrogen evolution, weight loss, PDP and EIS results verifies that GUE, PDE and TOE are effective in inhibiting the corrosion of AZ91 alloy in 3.5 wt% NaCl solution.

**Figure 4:**
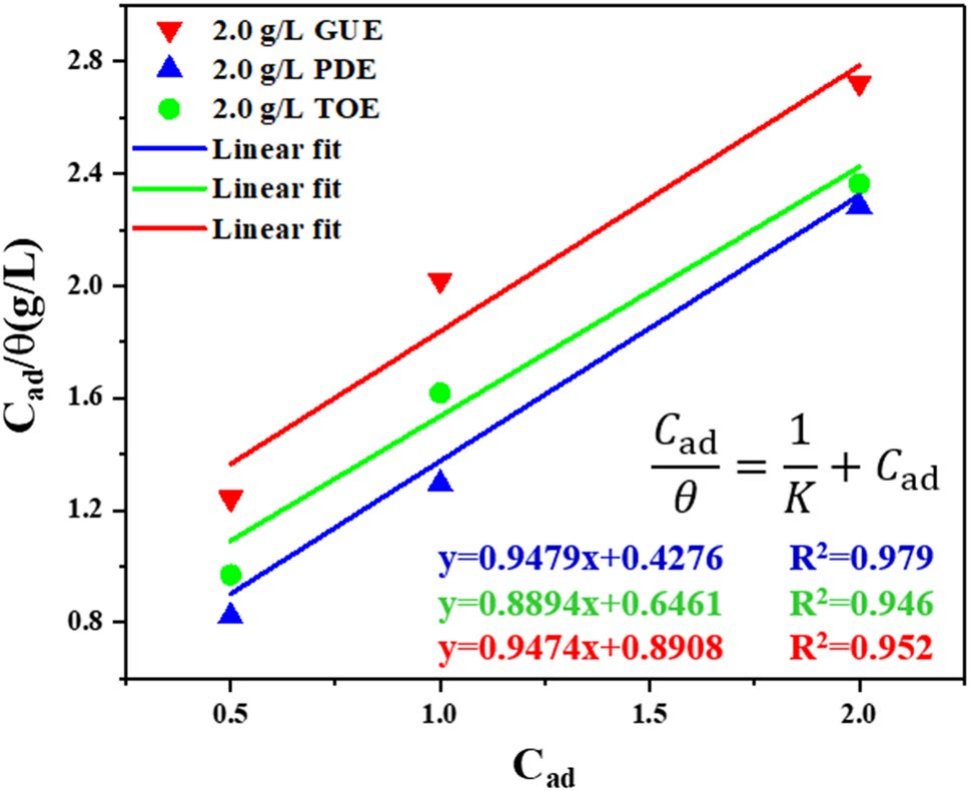
Langmuir adsorption isotherm for adsorption of GUE, PDE and TOE on AZ91 alloy.

As mentioned above, the corrosion inhibition is primarily attributable to the adsorption of TCM inhibitors onto the metal surface. In this scenario, the metal surface covered by inhibitors is resistant to corrosion while the exposed one is vulnerable to corrosion. Therefore, the degree of surface coverage θ can be used to analyze the adsorption behavior of TCM inhibitors, which can be quantified by adsorption isotherm models^44^. The fitting results revealed that all the inhibitors GUE, PDE and TOE obey Langmuir isotherm. i.e.

8 $$ \frac{{\text{C}}_{\text{ad}}}{{\uptheta }} = \frac{1}{{\text{k}}} + {{\text{C}}_{\text{ad}}} $$

where C_ad_ is the inhibitor concentration,${\text{k}}$ is the equilibrium constant of the adsorption–desorption process and θ defined as η was obtained from gravimetric experiment. Figure 4 illustrates the plot of C_ad_/θ against C_ad_. In terms of GUE, PDE and TOE, their values of linear correlation coefficient R^2^ are 0.952, 0.979 and 0.946, respectively, which are approximately equal to 1. This indicates that the adsorption of GUE, PDE and TOE on AZ91 alloy surface follows Langmuir isotherm^44^. It is noteworthy that the fitting results have a little deviation from the ideal Langmuir isotherm model, in which the interaction among adsorbed inhibitor molecules is assumed not to occur. However, as suggested by Alhaffar et al., the organic inhibitors with polar groups in their molecular structure would mutually repulse or attract, affecting the adsorption effect^38^.

### SEM and EDS analysis

**Figure 5:**
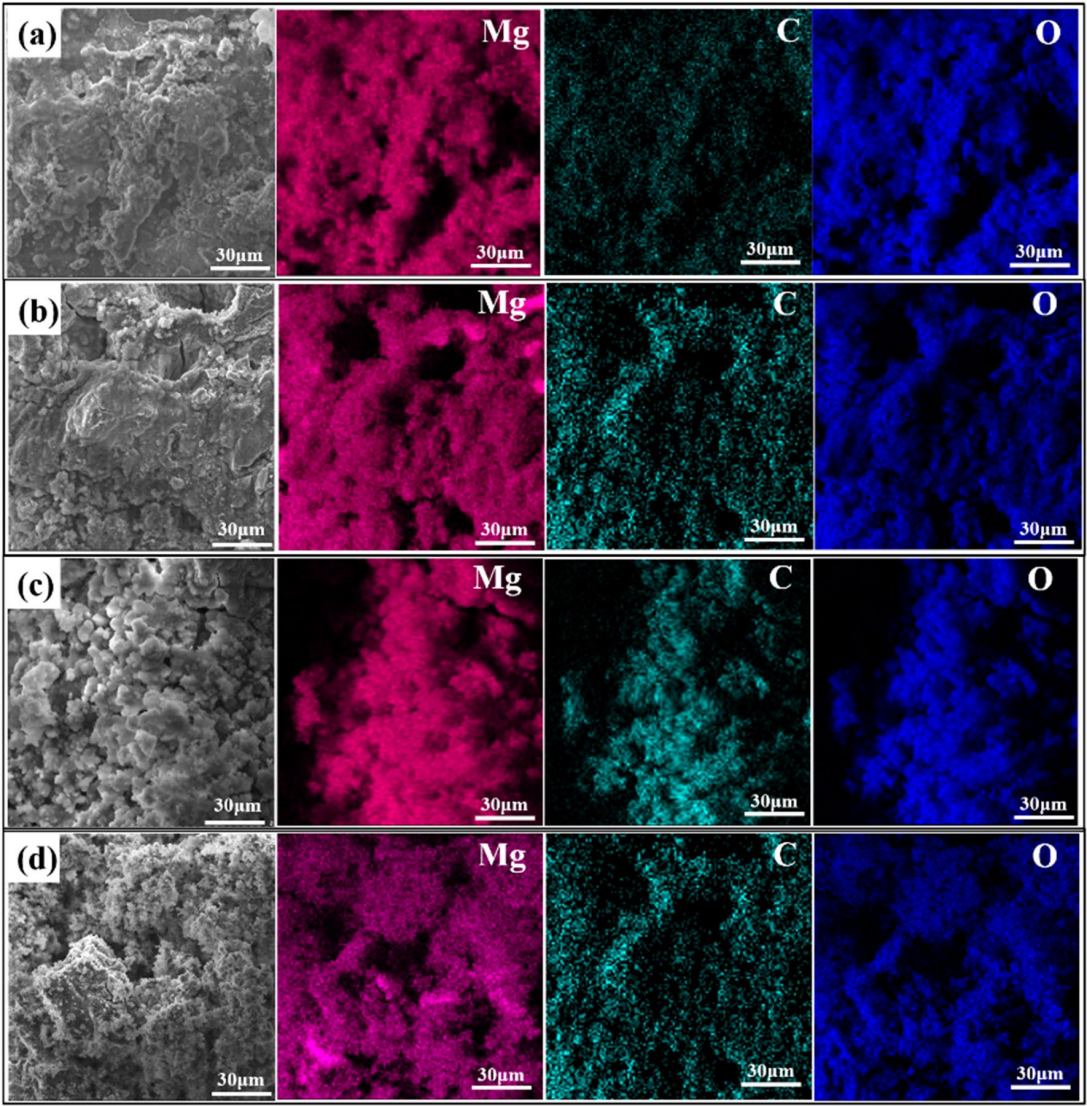
SEM and EDS analysis of AZ91 Mg alloys immersed in 3.5 wt% NaCl solution (**a**) without and with (**b**) 2 g/L GUE (**c**) 2 g/L PDE (**d**) 2 g/L TOE for 24 h under normal atmospheric condition.

The surface morphology of AZ91 alloy immersed in 3.5 wt% NaCl solution with and without GUE, PDE and TOE for 24 h is characterized by SEM and EDS, and the results are presented in Fig. 5. For accuracy, the SEM images with lower magnification are also provided and exhibited in Fig. S1. The corrosion products formed on the AZ91 alloy surface appeared to be loose and incomplete (Fig. S1(a)) but tended to be smoother after addition of GUE, PDE and TOE (Fig. S1(b, c, d)), suggesting that the substrate was covered by a protective film and thus slightly corroded. The EDS analysis in Fig. 5 shows that the alloy surfaces in all solutions are mainly composed of Mg, O and C. Previous research suggested that the main corrosion product of Mg and its alloys in neutral medium is Mg (OH)_2_^18,24,25^. However, the Mg (OH)_2_ precipitate has a loose and porous structure such that the alloy surface is inclined to be heavily corroded due to its extremely limited protection effect, leading to a rough and porous surface of AZ91 alloy. From the EDS mapping results, the C element concentration was increased in the presence of three TCM inhibitors compared with blank, as shown in Fig. 5b-d. It can be reasonably inferred that the adsorption of TCM inhibitors onto the alloy surface could facilitate the formation of a C containing protective film and thus a smooth and homogeneous alloy surface was obtained.

### LCM analysis

**Figure 6:**
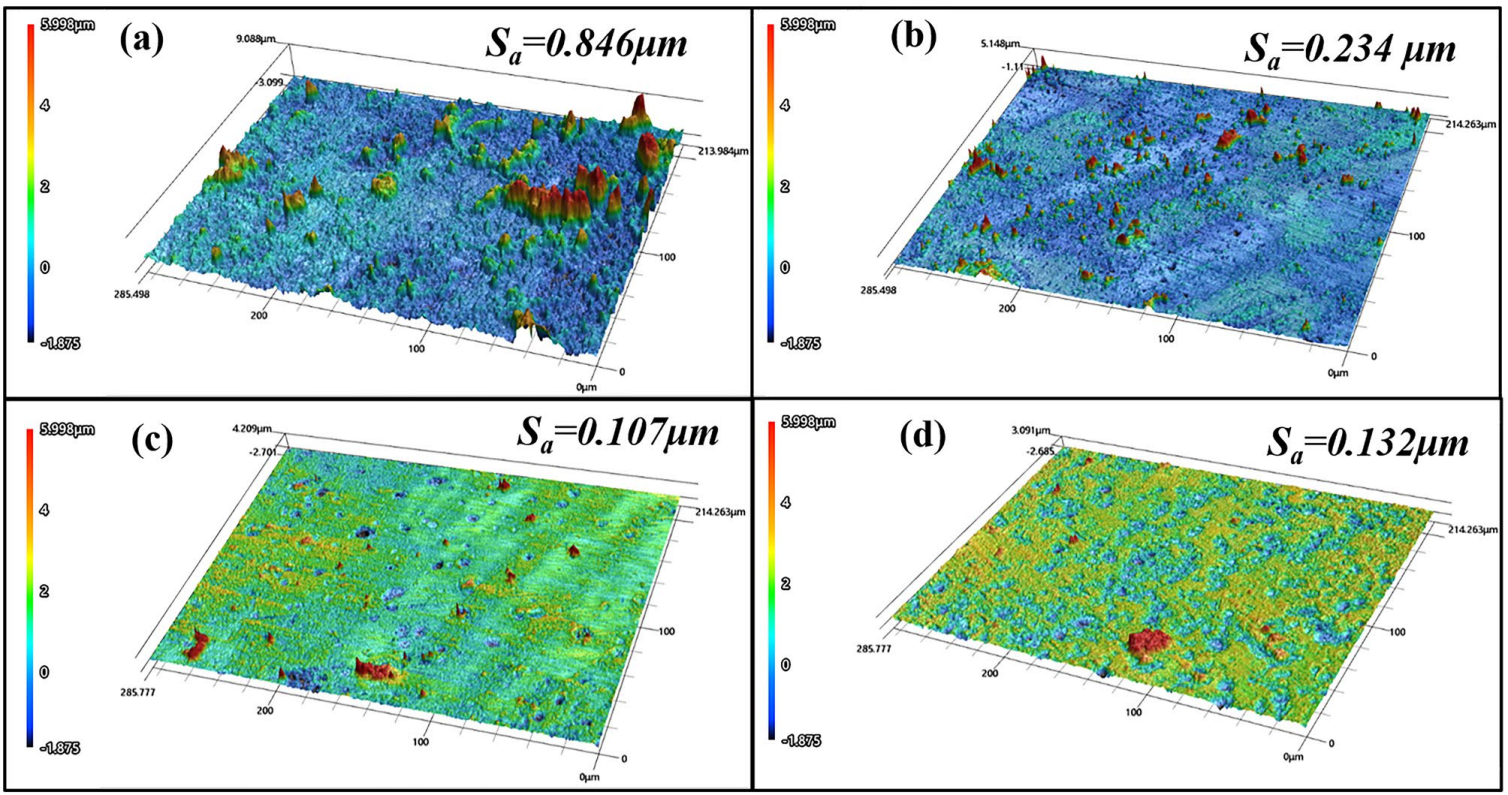
Roughness analysis of surface of AZ91 Mg alloys after immersing in 3.5 wt% NaCl solution (**a**) without and with addition of (**b**) 2 g/L GUE (**c**) 2 g/L PDE (**d**) 2 g/L TOE for 24 h under normal atmospheric condition.

The surface structure of AZ91 alloy samples immersed in 3.5 wt% NaCl solution without (a) and with GUE (b), PDE (c) and TOE (d) for 24 h under normal atmospheric condition were investigated by LCM analysis and the typical 3D images are presented in Fig. 6. As shown in the 3D image in Fig. 6a, a rugged surface structure can be clearly observed for the blank sample, revealing a heavily corroded surface with a roughness of 0.846 μm from the NaCl solution. When it comes to the case of TCM inhibitor containing solution, there is a clear reduction in the number and height of peaks on the sample surface. Compared with GUE and TOE, the addition of PDE can give rise to the smoothest surface with the least number of peaks and valleys existing on the surface. By measurements, the surface roughness values of samples immersed in NaCl solution with addition of GUE, PDE and TOE are 0.234, 0.107 and 0.132 μm, respectively, which are 72.34, 87.35 and 84.39% lower than that of blank one. It can be safely concluded that the TCM inhibitors adsorb onto the alloy surface and form a protective film to effectively isolate the corrosive species from the surface, thus inducing a remarkable inhibiting effect. By comparison with GUE and TOE, PDE evinces more excellent anti-corrosion performance on AZ91 alloy in 3.5 wt% NaCl solution, which is concordant with the electrochemical and SEM results.

## Discussion

### FTIR and UV analysis

**Figure 7:**
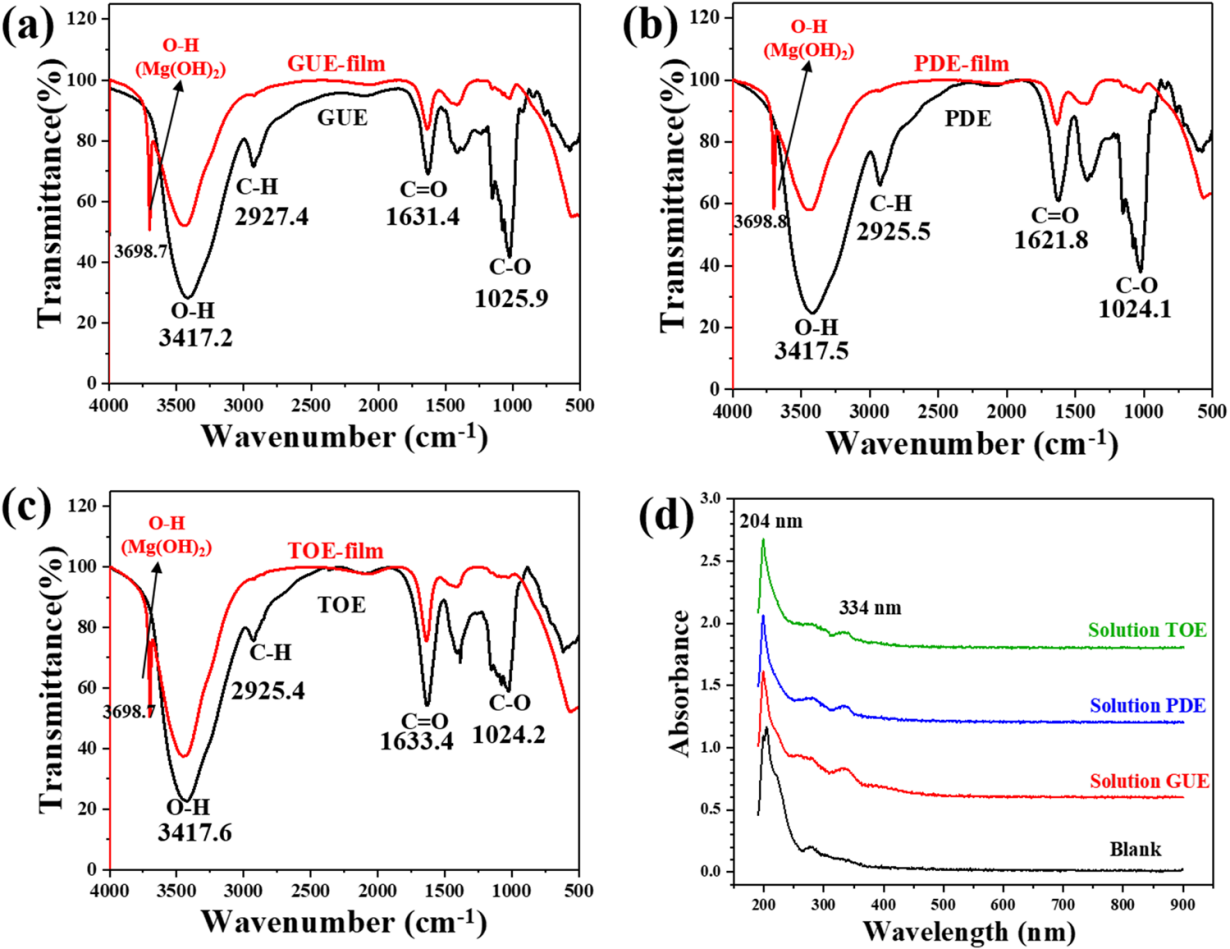
(**a**–**c**) show the FTIR spectra for pure GUE, PDE, TOE and the absorbed products extracted from the substrate surfaces after immersing in 3.5 wt% NaCl solution containing GUE, PDE, TOE, respectively. (**d**) UV–vis of 3.5 wt% NaCl solution devoid of and containing GUE, PDE, TOE after 24 h of immersing a AZ91 Mg alloy sample.

To unveil the adsorption behavior of TCM molecules on AZ91 alloy surface, FTIR experiment was performed and the results are shown in Fig. 7. In the pure GUE spectrum as exhibited in Fig. 7a, there are basically four main adsorption peaks that are located at 1025 cm^−1^, 1631 cm^−1^, 2927 cm^−1^ and 3417 cm^−1^, respectively. The characteristic peak at 1025 cm^−1^ may be associated with C–O stretching vibration^4^. In addition, the peaks at 1631 cm^−1^ and 2927 cm^−1^ may be related to the C = O and C-H stretching vibrations respectively, whereas the peak at 3417 cm^−1^ may be attributed to the O–H stretching vibration^45^. In the FTIR spectrum of GUE-film, the C–H and C–O stretching peaks were almost absent and an additional peak emerged at 3698 cm^−1^, which is a characteristic one of Mg(OH)_2_^46^. Similar phenomena can be found in the FTIR spectra of PDE and TOE. The FTIR analysis indicates that the adsorption behavior of GUE, PDE and TOE would occur by the interaction between the hydroxide/carboxide and the alloy surface.

The UV–Vis spectra of 3.5 wt% NaCl solution with and without GUE, PDE and TOE after immersion of the AZ91 alloy sample for 24 h are presented in Fig. 7d. From the blank solution spectrum, it can be observed that there is a strong adsorption band centered at 204 nm, which is attributable to the formation of hydroxide^47^. This further corroborates the presence of Mg (OH)_2_ in the corrosion products as evidenced in the FTIR result in Fig. 7a–c. Apart from the strong band at 204 nm, a weak band at 334 nm can also be detected in the spectra of the GUE, PDE and TOE containing solutions. The presence of new band at 334 nm confirms that Mg-inhibitor complexes could be formed on the AZ91 alloy surface. As indicated in literature, such complex can impede the anodic and cathodic reactions, leading to enhancement of corrosion mitigation.

### Analysis of Volta potential

**Figure 8:**
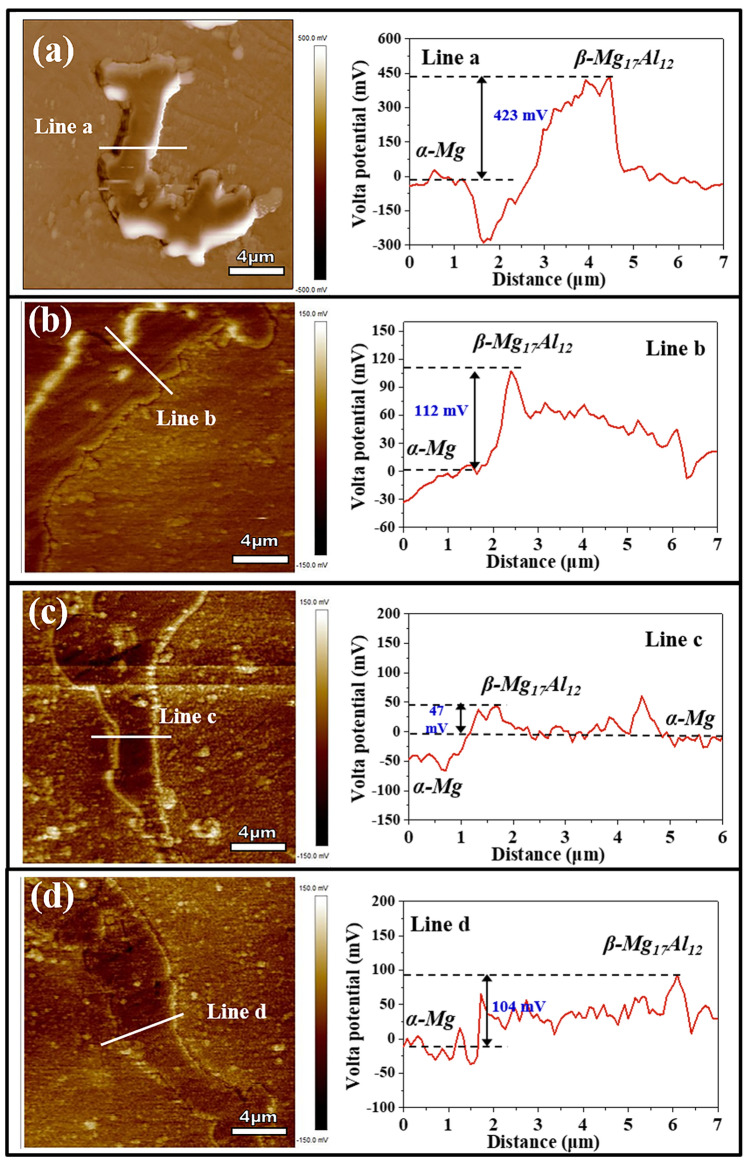
The surface volta potential maps of AZ91 alloys and corresponding potential profile of marked line after immersing in 3.5 wt% NaCl solution (**a**) without and with addition of (**b**) 2 g/L GUE (**c**) 2 g/L PDE (**d**) 2 g/L TOE for 24 h under normal atmospheric condition.

Essentially, the galvanic corrosion between cathodic β-Mg_17_Al_12_ and anodic α-Mg is mainly responsible for the degradation of AZ91 alloy, and the galvanic corrosion rate is highly dependent on the potential difference between the two phases. Therefore, it is of great necessity to investigate the potential difference between β-Mg_17_Al_12_ and α-Mg, especially influenced by the adsorption of GUE, PDE and TOE molecules. Figure 8 exhibits the SKPFM measurement of the AZ91 alloy before and after immersion in 3.5 wt% NaCl solution containing 2.0 g/L GUE, PDE and TOE for 24 h. The peak and valley located in the potential profile of marked line in Fig. 8a correspond to β-Mg_17_Al_12_ and α-Mg, respectively. It is apparent that the potential of β-Mg_17_Al_12_ is more noble than that of α-Mg, which is in accordance with the literature^48^. The potential difference of 423 mV between β-Mg_17_Al_12_ and α-Mg manifests a high corrosion rate of AZ91 alloy and thus a poor corrosion performance. After immersing AZ91 alloy into the NaCl solutions containing GUE, PDE and TOE, the potential difference between β-Mg_17_Al_12_ and α-Mg is greatly decreased from 423 mv to 112 mV, 47 mV and 104 mV, as exhibited in Fig. 8b,c,d, respectively. It is implied that the addition of TCM inhibitors leads to a significant reduction in the galvanic corrosion rate of AZ91 alloy. The lowest difference value of 47 mV means the lowest corrosion rate of AZ91 alloy and the highest inhibiting efficiency of PDE, which is in good agreement with the PDP, EIS and Hydrogen evolution experiments. Based on the SKPFM and FTIR analysis, it can be reasonably speculated that the adsorption of TCM molecules onto the surface of AZ91 alloy via hydroxyl and/or carbanyl functional groups present in TCM extracts may have a positive effect in reducing the potential difference between β-Mg_17_Al_12_ and α-Mg. As a consequence, the degradation of AZ91 alloy can be effectively mitigated, resulting in an improved corrosion resistance of AZ91 alloy.

### Inhibition mechanisms of TCM extracts

**Figure 9:**
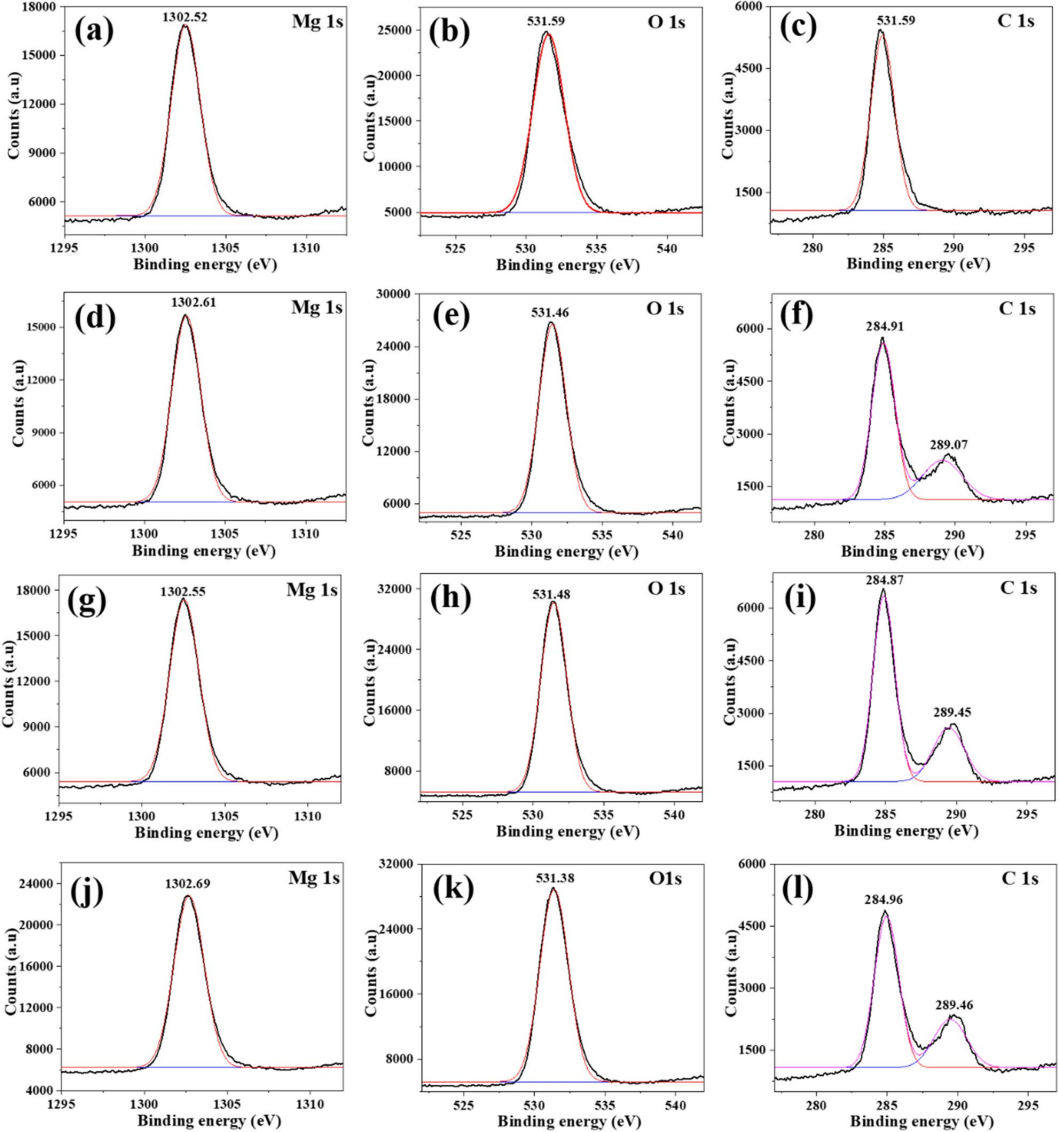
High-resolution spectra of film formed on AZ91 Mg alloy immersed in 3.5 wt% NaCl solution (**a**-**c**) without and with (**d**-**f**) GUE, (**g**-**i**) PDE, (**j**-**l**) TOE for 24 h at normal atmospheric condition.

To in-depth understand the inhibition mechanism of GUE, PDE and TOE, the AZ91 alloy surfaces after 24 h of immersion in 3.5 wt% NaCl solution with and without GUE, PDE and TOE were analyzed using XPS and the obtained results are shown in Fig. 9. The Mg 1 s peak at 1302.5 eV in Fig. 9a could be associated to Mg(OH)_2_, verifying that the corrosion product film on AZ91 alloy surface is mainly composed of Mg(OH)_2_ in the blank solution^49^. The XPS result is in accordance with those of FTIR and UV–Vis. As for the O 1 s spectra, the peaks at 531.6 eV, 531.5 eV, 531.5 eV and 531.8 eV in Fig. 9b,e,h,k, respectively, could be linked to the O–H group^40^. The corrosion of Mg alloy in an aqueous environment can be divided into anodic dissolution of Mg^50^:

9 $$ {\text{Mg}} \to {\text{Mg}}^{ + } + {\text{e}}^{ - } ,\;{\text{or}}\;{\text{Mg}}^{ + } \to {\text{Mg}}^{2 + } + {\text{e}}^{ - } $$

and cathodic hydrogen evolution:

10 $$ 2{\text{H}}_{2} {\text{O}} + 2{\text{e}}^{ - } \to 2{\text{OH}}^{ - } + {\text{H}}_{2} ,\;{\text{or}}\;2{\text{H}}^{ + } + 2{\text{e}}^{ - } \to {\text{H}}_{2} $$

Mg^2+^ ions can react with OH^-^ ions to form the main corrosion product, i.e. Mg (OH)_2_:

11 $$ 2{\text{Mg}}^{ + } + 2{\text{OH}}^{ + } \to {\text{Mg}}\left( {{\text{OH}}} \right)_{2} $$

**Figure 10:**
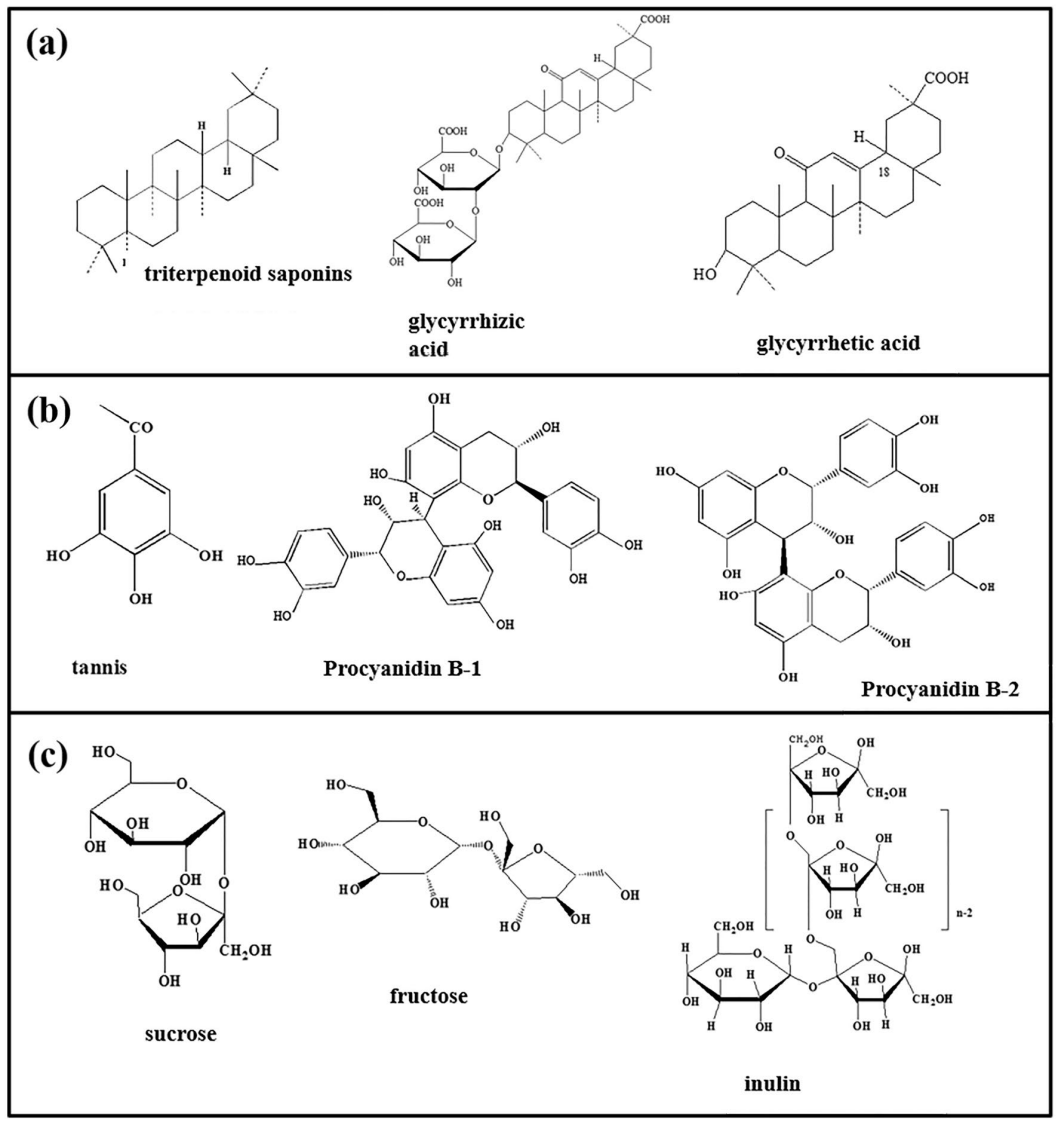
Molecular structures of the main components of (**a**) GUE, (**b**) PDE and (**c**) TOE, respectively.

Figure 9c shows the high-resolution spectrum of C 1 s in the blank solution. A single peak can be observed at 284.8 eV and could be attributed to C–C/C–H groups^51^. Unlike the C 1 s spectrum in Fig. 9c, additional C 1 s peaks can be found at 289.1 eV, 289.5 eV and 289.5 eV in Fig. 9f,I,l, respectively, which may correspond to the C=O group of the TCM inhibitors^52^. The XPS results combined with the FTIR analysis demonstrates that the interaction between the alloy surface and the C=O and/ or O–H groups from the organic components of inhibitors can promote the formation of a protective film. Research shows that *Glycyrrhiza uralensis* primarily consists of triterpenoid saponins, typically including glycyrrhizic acid (C_42_H_62_O_16_) and glycyrrhetic acid (C_30_H_46_O_4_), which contain carbanyl and hydroxyl functional groups^47^; Potentilla discolor is mainly composed of tannins, typically including Procyanidin B-1 (C_30_H_26_O_12_) and Procyanidin B-2 (C_30_H_26_O_12_) with a large quantity of polyphenols in their molecules^53^; *Taraxacum officinale* is constituted of polysaccgarides, typically including inulin (C_228_H_382_O_191_) and Fructose (C_6_H_12_O_6_), which possess a large number of hydroxyl functional groups^54^. The specific molecular structures of the main organic components of GUE, PDE and TOE are shown in Fig. 10. These compounds that often contain carbanyl, hydroxyl or polyphenols functional groups in their molecular structures are able to facilitate the adsorption of GUE, PDE and TOE onto AZ91 alloy surface, reducing the porosity of the original Mg (OH)_2_ film and thus retarding the degradation of the AZ91 alloy.

**Figure 11:**
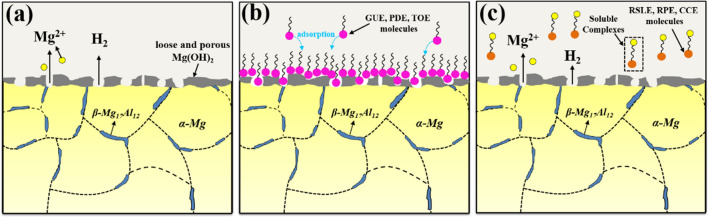
Schematic diagrams of corrosion mechanisms of AZ91 alloy in 3.5 wt% NaCl solution without and with TCM extracts. (**a**) Blank solution; (**b**) with addition of GUE, PDE or TOE; (**c**) with addition of RSLE, RPE or CCE.

Based on the analysis above, the TCM-induced inhibition mechanisms can be proposed to elucidate the corrosion behavior of AZ91 alloy in 3.5 wt% NaCl solution. Figure 11 shows the schematic diagrams of corrosion mechanisms of AZ91 alloy in NaCl solution with and without TCM extracts. In the initial stage of corrosion, the corrosion product of Mg(OH)_2_ would precipitate and form a loose and porous Mg(OH)_2_ layer on the surface of AZ91 alloy, because of which AZ91 alloy is highly susceptible to corrosion, as shown in Fig. 11a. Subsequently, the TCM (GUE, PDE and TOE) extract molecules could interact with the alloy surface via hydroxyl and/or carbanyl functional groups, resulting in the adsorption onto the alloy surface of the organic compounds contained in TCM extracts. The adsorption of multi-components in TCM extracts can promote the formation of a dense and continuous protective film, which can function as a robust barrier to prevent the penetration of corrosive chloride ions and water into the alloy surface, as exhibited in Fig. 11b. As mentioned above, GUE, PDE and TOE can be adsorbed on the anodic active sites to impede the anodic dissolution of Mg, more effectively retarding the anodic reaction. In addition, the adsorption of TCM inhibitors on the surface of AZ91 alloy can lead to a significant reduction in the potential difference between β-Mg_17_Al_12_ and α-Mg, thus greatly inhibiting the galvanic corrosion and improving the corrosion performance of AZ91 alloy. On the contrary, it is revealed from the experimental results that RSLE, RPE and CCE exhibit the negative inhibition effect compared with other extracts. The corrosion increased by RSLE, RPE and CCE might be attributed to the formation of soluble complexes between Mg^2+^ and RSLE, RPE, CCE molecules^24^. As a result, the dissolution of Mg is accelerated and the overall corrosion of AZ91 alloy is aggravated. Therefore, they can not act as effective corrosion inhibitors but corrosion promoters.

## Conclusions

Seven TCM extracts including ZORE, RSLE, RPE, CCE, GUE, PDE and TOE were screened as corrosion inhibitors for AZ91 alloy in 3.5 wt% NaCl solution. It is demonstrated that only GUE, PDE and TOE could exhibit inhibiting effect, and when the concentrations of GUE, PDE and TOE reached up to 2.0 g/L, their inhibiting efficiency was maximized to 73.4%, 87.6% and 84.6%, respectively. The electrochemical analysis combined with the surface observation revealed that both GUE and PDE were mixed-typed inhibitors while TOE was an anodic inhibitor, and the inhibition occurred through the adsorption onto the alloy surface of the multi-components contained in GUE, PDE and TOE, which follows the Langmuir adsorption isotherm. The adsorbed layer of inhibitor molecules could serve as a protective film, preventing the corrosive attack from chloride ions and water, mitigating the galvanic corrosion, and thus enhancing the corrosion performance of AZ91 alloy.

## Supplementary Information

Supplementary Information.

## Data Availability

All data included in this study are available upon request by contact with the corresponding author.
