# Supplementary material for: Traditional Chinese medicine extracts as novel corrosion inhibitors for AZ91 magnesium alloy in saline environment
Source: Sci Rep. 2022 May 5;12:7367. doi: 10.1038/s41598-022-10900-x (PMC9072390; doi:10.1038/s41598-022-10900-x)
Supplement: Supplementary file 1 — Supplementary Information. [file 41598_2022_10900_MOESM1_ESM.pdf]

Traditional Chinese medicine extracts as novel corrosion inhibitors for AZ91 magnesium alloy in saline environment

Haonan Li [a,b], Min Fan[c,*], Kui Wang [a,b,*], Xiaolan Bian [c,*], Haiyan Jiang [a,b], Wenjiang Ding [a,b]

[a] National Engineering Research Center of Light Alloy Net Forming, Shanghai Jiao Tong University, 200240, Shanghai, P.R. China.

[b] School of Materials Science and Engineering, Shanghai Jiao Tong University, 200240, Shanghai, P.R. China.

[c] Department of Pharmacy, Ruijin Hospital, School of Medicine, Shanghai Jiao Tong University, Shanghai 200003, China;

* Corresponding authors. E-mail addresses: fm04197@rjh.com.cn. (Min Fan), fateratory@sjtu.edu.cn (Kui Wang), bxl40338@rjh.com.cn (Xiaolan Bian).

Supplementary Figure

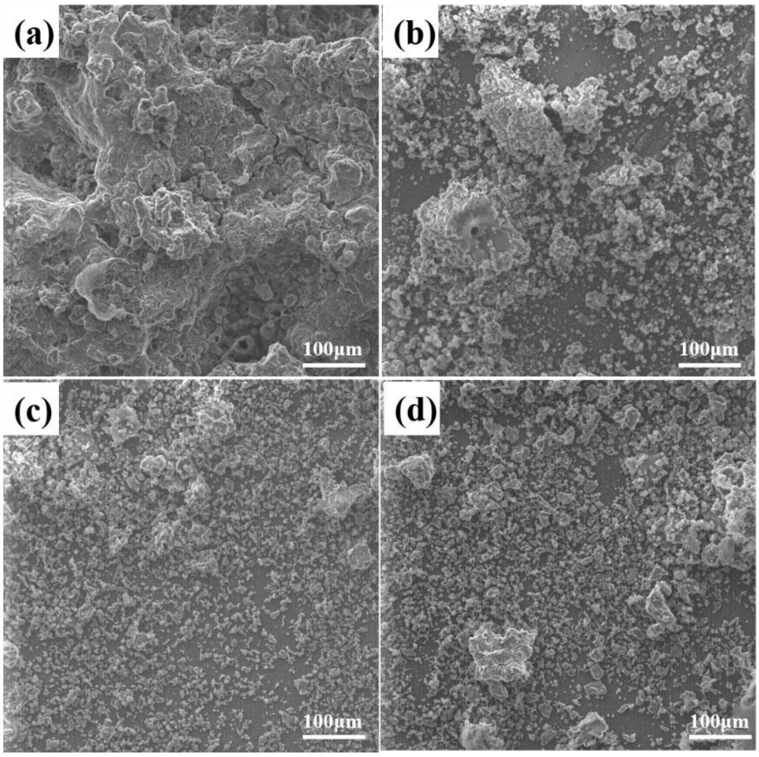

Figure S1. SEM images of surfaces of AZ91 Mg alloys immersed in 3.5 wt.% NaCl solution (a) without and with (b) 2g/L GUE (c) 2g/L PDE (d) 2g/L TOE for 24h under normal atmospheric condition.
